# Thalamic Circuit Diversity: Modulation of the Driver/Modulator Framework

**DOI:** 10.3389/fncir.2015.00086

**Published:** 2016-01-12

**Authors:** Martha E. Bickford

**Affiliations:** Department of Anatomical Sciences and Neurobiology, University of LouisvilleLouisville, KY, USA

**Keywords:** dorsal lateral geniculate nucleus, pulvinar nucleus, corticothalamic, thalamocortical, lateral posterior nucleus, retinogeniculate, tectothalamic

## Abstract

The idea that dorsal thalamic inputs can be divided into “drivers”, which provide the primary excitatory drive for the relay of information to cortex, and “modulators”, which alter the gain of signal transmission, has provided a valuable organizing principle for the study of thalamic function. This view further promoted the identification of “first order” and “higher order” thalamic nuclei, based on the origin of their driving inputs. Since the introduction of this influential terminology, a number of studies have revealed the existence of a wide variety of thalamic organizational schemes. For example, some thalamic nuclei are not innervated by typical driver inputs, but instead receive input from terminals which exhibit features distinct from those of either classic drivers or modulators. In addition, many thalamic nuclei contain unique combinations of convergent first order, higher order, and/or other “driver-like” inputs that do not conform with the driver/modulator framework. The assortment of synaptic arrangements identified in the thalamus are reviewed and discussed from the perspective that this organizational diversity can dramatically increase the computational capabilities of the thalamus, reflecting its essential roles in sensory, motor, and sensory-motor circuits.

## Universal Features of Thalamic Circuits

**Figure 1:**
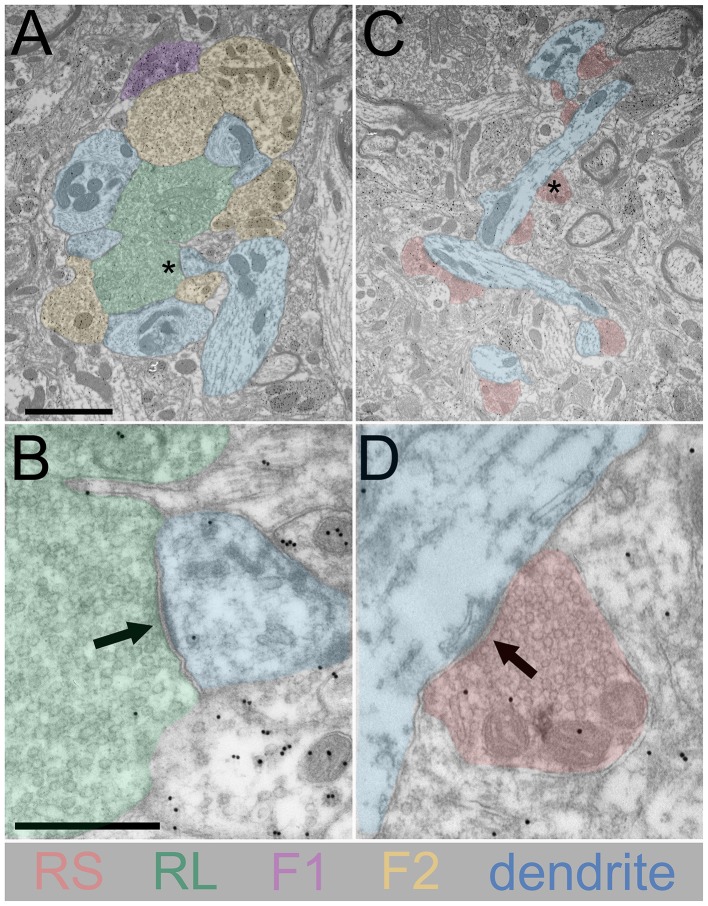
**Synaptic terminal types in the dorsal thalamus defined using electron microscopy**. Electron microscopic images of the cat dorsal lateral geniculate nucleus (dLGN) are shown. Tissue was obtained from a previous study (Bickford et al., 2008); postembedding immunocytochemical techniques were used to reveal the presence of gamma amino butyric acid, GABA, with gold particles). **(A)** A dLGN glomerulus is illustrated which contains a large profile with round vesicles (RL, green), GABAergic dendritic terminals (F2, yellow, high density of gold particles), and relay cell dendrites (blue). A GABAergic axon terminal (F1, purple, high density of gold particles) is located at the periphery of the glomerulus. The asterisk indicates the location of a synapse shown at higher magnification in **(B)**. **(B)** The arrow indicates a synaptic contact of the RL profile (green) onto a relay cell dendrite (blue). **(C)** A non-glomerular region of the dLGN is illustrated which contains small profiles with round vesicles (RS, pink) that synapse on relay cell dendrites (blue). The asterisk indicates the location of a synapse shown at higher magnification in **(D)**. **(D)** The arrow indicates the synaptic contact of an RS profile (pink) onto a relay cell dendrite (blue). Scale in **(A)** = 2 μm and also applies to **(C)**. Scale in **(B)** = 0.5 μm and also applies to **(D)**.

Early electron microscopic studies of the dorsal thalamus revealed a number of similarities across sensory-related nuclei. Studies of the dorsal lateral geniculate nucleus (dLGN; Szentagothai, 1963; Guillery, 1969; Pasik et al., 1973), ventrobasal nucleus (VB; Ralston and Herman, 1969), medial geniculate nucleus (MGN; Majorossy and Réthelyi, 1968), and pulvinar nucleus (Majorossy et al., 1965; Mathers, 1972a) demonstrated the presence of complex (glomerular, Figure 1A) synaptic arrangements in which large synaptic terminals that contain round vesicles (RL profiles, Figures 1A,B; green) contact the proximal dendrites of thalamocortical relay cells (Figures 1A,B; blue), as well as the dendritic terminals of interneurons which contain sparsely distributed flattened or pleomorphic vesicles (F2 profiles, Figure 1A; yellow). RL profiles were identified as arising from the retina (axons traveling in the optic tract to the dLGN; Szentagothai, 1963), trigeminal nucleus (medial lemniscus to the VB; Ralston, 1969), inferior colliculus (lateral lemniscus to the MGN; Majorossy and Réthelyi, 1968) or cortex (internal capsule to the pulvinar; Mathers, 1972b). Two additional terminal types were identified across thalamic nuclei: small terminals that contained round vesicles (RS profiles, Figures 1C,D; red) that primarily contact the more distal portions of relay cell dendrites (Figures 1C,D; blue), and terminals that contained a high density of flattened vesicles (F1 profiles, Figure 1A; purple).

## The Driver/Modulator Concept

The identified similarities in sensory thalamus circuits led Sherman and Guillery (1998) to propose an organizing framework of thalamic circuitry that has inspired numerous studies and greatly advanced our understanding of thalamic function. Based on the finding that the receptive field properties of dLGN neurons are nearly identical to that of their retinal inputs (Cleland et al., 1971), as well as the finding that each dLGN cell is innervated by only a few retinal ganglion cell axons (Hamos et al., 1987). Sherman and Guillery (1998) proposed that the receptive field properties of each thalamic nucleus are determined by RL inputs that originate from a single source. In the dLGN, although retinal input comprises only 5–10% of the synapses (Van Horn et al., 2000), it is nevertheless the primary determinate of geniculate activity, and is therefore aptly named the driving input. Within this framework, RL inputs across the thalamus are proposed to drive activity patterns (i.e., determine receptive field properties), while the remaining inputs to each nucleus are considered modulators, which can alter the transmission of sensory-driven activity in a state-dependent manner.

The prime examples of modulating inputs are the RS profiles, which in the dLGN, are either glutamatergic inputs that originate from layer VI of the striate cortex (Gilbert and Kelly, 1975), or cholinergic/nitrergic terminals that originate from the pedunculopointine tegmentum (PPT; Bickford et al., 1993; Erişir et al., 1997a; Erişir et al., 1997b). Both of these RS inputs have been found to influence the responsiveness of geniculate neurons, without dramatically changing their receptive field properties. Stimulation of the PPT increases the responsiveness of geniculate neurons to their driving retinal inputs (Lu et al., 1993), providing a mechanism for the global regulation of visual signal transfer during different states of arousal. Corticothalamic inputs may additionally tune activity patterns to enhance the responsiveness of restricted populations of thalamic neurons to their driving inputs, thereby aligning the actions of the thalamus and cortex (Briggs and Usrey, 2008).

## Biophysical Features of Drivers and Modulators

**Figure 2:**
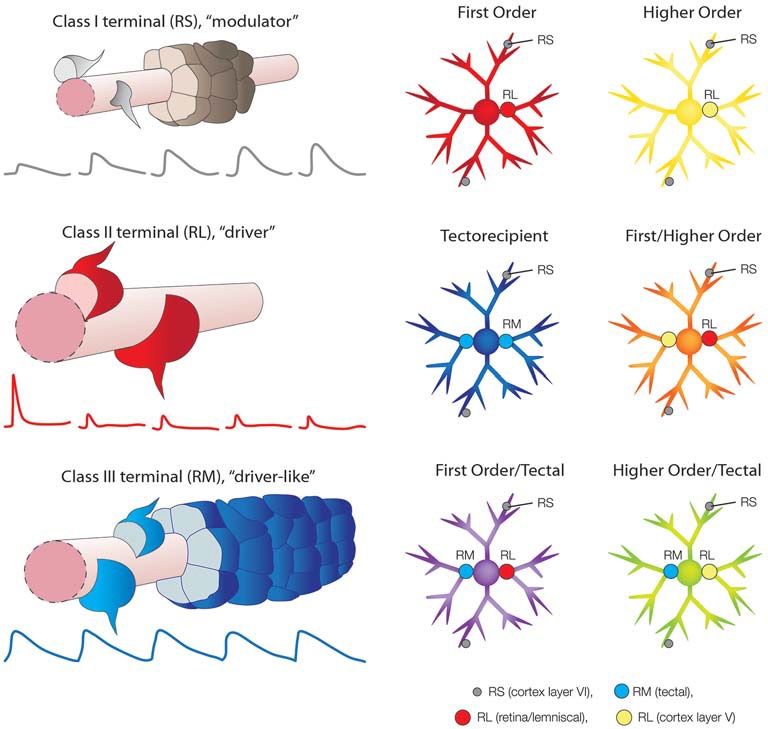
**Schematic summary of synaptic terminals types and their arrangements in the dorsal thalamus.** *Class I* axons (Guillery, 1966) form small terminals with round vesicles (RS; Guillery, 1969) that are defined as modulators (Sherman and Guillery, 1998). RS terminals that originate from cortex layer VI converge on small caliber (distal) dendrites (depicted by the gray terminals surrounding a section of dendrite, pink, modified from Robson and Hall, 1977). Repetitive stimulation of layer VI corticothalamic terminals results in a frequency-dependent facilitation of excitatory postsynaptic potentials (EPSPs; depicted by gray traces, from Li et al., 2003a). *Class II* axons (Guillery, 1966) form large terminals that contain round vesicles (RL; Guillery, 1969) that are defined as drivers (Sherman and Guillery, 1998). RL terminals that originate from the retina, medial or lateral lemniscus, or cortex layer V, form relatively few synapses on large caliber (proximal) dendrites (depicted by the red terminals surrounding a section of dendrite, pink). Repetitive stimulation of RL terminals results in a frequency-dependent depression of excitatory postsynaptic potentials (depicted by red traces, from Li et al., 2003a). *Class III* terminals form medium size terminals that contain round vesicles (RM; Robson and Hall, 1977), here refered to as “driver-like”. RM terminals that originate from the superior colliculus (tectal) converge on large caliber (proximal) dendrites (depicted by the blue terminals surrounding a section of dendrite, pink, modified from Robson and Hall, 1977). Repetitive stimulation of tectothalamic terminals results in little change in the amplitude of EPSPs (depicted by blue traces, from Masterson et al., 2010). *First order* nuclei (red neuron, modified from Bickford et al., 2015) receive a small number of RL inputs on their proximal dendrites that originate from a single subcortical source (red terminal). *Higher order* nuclei (yellow neuron) receive a small number of RL inputs on their proximal dendrites that originate from cortex layer V (yellow terminal). *Tectorecipient* nuclei (dark blue neuron) receive convergent RM inputs on their proximal dendrites (light blue terminals). As discussed in the text, a variety of combinations of first order, higher order and tectal inputs have been identified which may result in emergent receptive field properties (depicted by the orange, purple and green neurons).

RL profiles are approximately 10 times larger than RS profiles (Li et al., 2003b; Bickford et al., 2010, 2015), and each RL bouton establishes numerous synaptic contacts (Budisantoso et al., 2012; Hammer et al., 2014, 2015), whereas RS profiles typically form single synapses with their postsynaptic partners (Jones and Powell, 1969; Erişir et al., 1997b). *In vitro* studies of responses elicited by activation of retinogeniculate or corticothalamic terminals in brain slices revealed that RL and RS profiles evoke very distinct types of postsynaptic responses. RL terminals exhibit a high probability of neurotransmitter release and their stimulation initially elicits large amplitude, fast, primarily ionotropic, glutamatergic responses; repetitive stimulation of RL profiles depletes synaptic vesicles and desensitizes postsynaptic receptors so that the amplitudes of postsynaptic responses rapidly decrease in a frequency-dependent manner (Figure 2 *class II, RL profile*, driver, red traces; Turner and Salt, 1998; Chen and Regehr, 2003; Li et al., 2003a; Reichova and Sherman, 2004; Groh et al., 2008; Budisantoso et al., 2012). In contrast, stimulation of RS corticothalamic terminals initially elicits smaller amplitude, inotropic glutamatergic responses. These terminals exhibit a low probability of glutamate release, but their repetitive stimulation rapidly increases the amplitudes of postsynaptic responses in a frequency-dependent manner (Figure 2 *class I, RS profile, modulator*, gray traces; Turner and Salt, 1998; Granseth et al., 2002; Kielland et al., 2006; Jurgens et al., 2012). Repetitive stimulation of corticothalamic terminals can also activate metabotropic glutamate receptors (McCormick and von Krosigk, 1992). Finally, electrical stimulation of layer VI corticothalamic axons with increasing current levels results in a graded increase in the amplitude of postsynaptic responses, demonstrating that many RS terminals converge on postsynaptic neurons (Figure 2 *class I, RS profile, modulator*, gray; Li et al., 2003a, b; Masterson et al., 2009, 2010). In contrast, electrical stimulation of RL axons with increasing current levels results in “all or none” changes in the amplitude of postsynaptic responses, demonstrating that each postsynaptic neuron receives input from only a few RL axons (Figure 2 *class II, RL profile, driver*, red; Li et al., 2003a, b; Ziburkus and Guido, 2006).

## First and Higher Order Thalamic Nuclei

A further organizing principal that grew from the driver/modulator framework of thalamic function was the ability to categorize nuclei based on the origin of their driving input. Sherman and Guillery (1998) defined first order nuclei as those that receive their driving input from sources that relay information from peripheral sensory receptors, such as the retinal input to the dLGN, or the leminiscal inputs to the VB and MGN (Figure 2; *first order*, red neuron). Higher order nuclei are defined as those that receive their driving input from the cortex, specifically from neurons in layer V (Figure 2; *higher order*, yellow neuron). The chief example of a higher order nucleus is the pulvinar nucleus, which receives very little ascending subcortical input (Rovó et al., 2012), but receives abundant input from corticothalamic cells located in both layer V and layer VI. In particular, the striate-recipient zones of the pulvinar nucleus (or lateral posterior nucleus, LPN, of carnivores and rodents) are the best examples of higher order thalamic nuclei (Mathers, 1972b; Ogren and Hendrickson, 1979; Abramson and Chalupa, 1985; Guillery et al., 2001; Li et al., 2003b; Huppé-Gourgues et al., 2006).

The idea that each thalamic nucleus is driven by a single primary input suggested that the function of higher order thalamic nuclei may be to transfer information from one cortical area to another. In other words, it has been suggested that the receptive field properties of pulvinar neurons are driven by layer V input from one cortical area, and these signals are transferred via the pulvinar to other cortical areas (Guillery and Sherman, 2002; Sherman and Guillery, 2002). While this hypothesis has not been fully tested with *in vivo* experiments, the existence of cortical-thalamo-cortical signal transmission has been demonstrated *in vitro* (Theyel et al., 2010).

## Tectorecipient Thalamic Nuclei and Spatial Integration

Although many thalamic nuclei can be categorized as first or higher order, it is now apparent that this nomenclature must be modified in order to include the wide variety of “non-canonical” thalamic circuits that have been identified in more recent years. For example, thalamic nuclei that are innervated by the superior colliculus cannot be classified as either first or higher order because, although tectothalamic synaptic terminals are not archetypal drivers, they are larger than all other synaptic terminals within these nuclei. Tectothalamic inputs can be considered “driver-like” in that they are medium-sized terminals that contain round vesicles (RM profiles) that innervate proximal dendrites (Figure 2 *class III, RM profile, driver-like*, blue; Robson and Hall, 1977; Kelly et al., 2003; Chomsung et al., 2008; Masterson et al., 2009) and release glutamate to activate ionotropic glutamate receptors on postsynaptic neurons (Masterson et al., 2010). However, unlike typical driver inputs, many tectothalamic inputs can converge on individual neurons, and in nuclei where this convergence occurs, stimulation of tectothalamic inputs at frequencies of up to 20 Hz elicits postsynaptic responses that maintain stable amplitudes (Figure 2 *class III, RM profile, driver-like*, blue traces). That is, tectothalamic inputs exhibit neither frequency-dependent depression, nor facilitation. However, stimulation at 100 Hz can elicit the release of substance P from these terminas which, through activation of neurokinin one receptors, can boost tectothalamic responses (Masterson et al., 2010). Finally, tectothalamic terminals contain a different complement of presynaptic proteins than those found in classic drivers or modulators (Wei et al., 2011). Thus, tectorecipient nuclei (Figure 2; *tectorecipient*, blue neuron) are distinct from either first or higher order nuclei, which both contain RL profiles.

The absence of RL inputs has been described in other thalamic nuclei (Smith et al., 2007; Rovó et al., 2012). In the paralaminar region of the MGN, inputs originating from the superior and inferior colliculi, were described as “integrators” (Smith et al., 2007). The idea behind this nomenclature is that within nuclei that lack typical driver inputs, the collective activity of many convergent inputs may determine the receptive field properties of thalamic neurons. Support for this concept was provided by Chalupa et al. (1983); who found that the receptive field sizes of neurons in the tectorecipient zone of the cat LPN were much larger than those of neurons in the superficial layers of the superior colliculus. This suggests that, in some regions of the thalamus, the convergence of multiple inputs onto individual neurons provides spatial integration to create unique, emergent, receptive field properties.

## First and Higher Order Convergence and Temporal Integration

Groh et al. (2014) clearly demonstrated the convergence of both first and higher order driver inputs onto single neurons in the somatosensory thalamus (Figure 2; *first order/higher order*, orange neuron). Using anatomical techniques, they demonstrated that large synaptic terminals from both the trigeminal nucleus and layer V of the barrel cortex innervated the proximal dendrites of single neurons in the medial subdivision of the mouse posterior nucleus. They then established that when activated simultaneously, these two inputs combine in a supralinear fashion. Such convergence therefore provides a mechanism for the synergistic amplification of signals within a narrow temporal window. In this case the convergence of two driver inputs may report the relative timing between sensory events and ongoing cortical activity.

## First Order and Tectal “Driver-Like” Convergence: Sensory/Motor Integration?

Even within the first order dLGN, where the synaptic arrangements originally inspired the driver/modulator framework, there are restricted regions that contain unique circuits. In the dorsolateral shell of the mouse dLGN, inputs from the superior colliculus and the retina were demonstrated to converge on single neurons using both anatomical and physiological approaches (Bickford et al., 2015; Figure 2; *first order/tectal*, purple neuron). In this case, such convergence may be used integrate visual and motor signals. For example the convergence of retinal and tectal inputs in the dLGN may be necessary to calculate the trajectory of visual stimuli in relation to movement of the body.

## Convergence of Higher Order and “Driver-Like” Inputs?

There are a number of thalamic regions that are innervated by large driver terminals that originate from the cortex, as well as ascending driver-like terminals. One region is the rodent LPN, where large terminals that originate from the primary visual cortex overlap the distribution of terminals that originate from the superior colliculus (Li et al., 2003b; Masterson et al., 2009). Another example is the cat pulvinar nucleus where large terminals that originate from cortical area 7 overlap the distribution of large terminals that originate from the pretectum (Baldauf et al., 2005a, b). Many other possible combinations have been revealed by the distributions of the type 1 and type 2 vesicular glutamate transporters, which are found in cortical and subcortical inputs respectively (Rovó et al., 2012). While the convergence of tectal/pretectal and higher order inputs onto single neurons has not yet been definitively demonstrated, the variety of terminal patterns found across the thalamus suggest that novel spatial and temporal receptive field properties can potentially be constructed via the integration of first order, higher order and/or other driver-like inputs.

## Additional Thalamic Diversity

This short review highlights just a few of the variations of the driver/modulator framework, by focusing on thalamic nuclei related to audition, somatosensation and vision. When the full complement of thalamic nuclei is considered, a host of additional synaptic arrangements can be identified. For example, nuclei of the motor thalamus receive convergent input from the cortex, cerebellum and basal ganglia, and have been described as “super integrators” (Bosch-Bouju et al., 2013). Finally, in addition to the various arrangements of glutamatergic inputs, a wide variety of inhibitory circuits have been identified that can provide potent suppression of thalamic activity (Barthó et al., 2002; Bokor et al., 2005; Bodor et al., 2008; Giber et al., 2015).

## Summary and Future Directions

The detailed study of thalamic circuits has unveiled a wide range of potential computational capabilities. Receptive field properties in both first and higher order nuclei are likey driven by a single input, and modulated in a state dependent manner. In contrast, receptive field properties in tectorecipient nuclei may be created by the integration of multiple convergent inputs. A wide array of additional thalamic receptive field properties may be created, dependent on the degree of convergence and relative timing of first order, higher order, and/or other driver-like inputs.

Correlations between the diversity of thalamic circuits and thalamocortical circuits may be a particularly fruitful avenue for furthering our understanding of thalamic function. As recently reviewed by Harris and Shepherd (2015); the division of the thalamus into “core” and “matrix” nuclei based on their thalamocortical projection patterns (Jones, 1998, 2001) is a useful starting point, in that the core and matrix categories roughly correlate with first and higher order nuclei. In primary sensory areas of cortex, the thalamocortical axons originating from core nuclei primarily target layer IV (e.g., core/first order dLGN projections to V1; Winfield and Powell, 1976; Winfield et al., 1982; Raczkowski and Fitzpatrick, 1990; Nahmani and Erisir, 2005; Familtsev et al., 2015), whereas thalamocortical axons originating from matrix nuclei target layers I and V (e.g., matrix/higher order pulvinar/LPN projections to V1; Ogren and Hendrickson, 1977; Carey et al., 1979; Herkenham, 1980; Abramson and Chalupa, 1985).

However, as stated by Harris and Shepherd (2015); “the concepts of core- and matrix-type projections may need to be extended to manage the full complexity of thalamic projections to higher order cortex”. Toward this end, Clascá et al. (2012) have described four categories of thalamic nuclei (core, matrix-focal, matrix-interareal, and intralaminar) to incorporate the diversity of thalamocortical projection patterns, as well as the subcortical projections of the thalamus to the striatum and amygdala. Within this framework, the matrix-focal category is typified by neurons in the koniocellular layers (primate), or shell (rodent) of the dLGN, which project to the superficial layers of V1 (Hendry and Reid, 2000; Shostak et al., 2002; Cruz-Martín et al., 2014; Bickford et al., 2015). The matix-intrareal category is correlated with nuclei such as the tectorecipient pulvinar or LPN, where neurons project to multiple visual areas, as well as the striatum and amygdala (Chomsung et al., 2010; Day-Brown et al., 2010; Nakamura et al., 2015).

Recent anatomical and optogenetic studies have demonstrated that thalamic axons can target a wide array of cortical cell types, dependent on the cortical area, cortical lamina, and thalamic nucleus of origin (Petreanu et al., 2009; Cruikshank et al., 2010, 2012; Hooks et al., 2013; Kloc and Maffei, 2014; Shigematsu et al., 2015). Thus a challenge for future studies will be the documentation and classification of thalamocortical microcircuits. As evidenced by the advancements achieved since the introduction of the driver/modulator framework, identification of canonical microcircuits is a key component in deciphering nervous system function. The subsequent identification of variations in standard circuit modules allows us then to build and expand upon these conceptual frameworks, driving the field forward.

## Author Contributions

MEB wrote the manuscript.

## Funding

This work was supported by the National Eye Institute (R01EY024173).

## Conflict of Interest Statement

The author declares that the research was conducted in the absence of any commercial or financial relationships that could be construed as a potential conflict of interest.
